# Barriers to recruitment, retention and intervention delivery in a randomized trial among patients with firearm injuries

**DOI:** 10.1186/s40621-021-00331-z

**Published:** 2021-07-26

**Authors:** Anthony S. Floyd, Vivian H. Lyons, Lauren K. Whiteside, Kevin P. Haggerty, Frederick P. Rivara, Ali Rowhani-Rahbar

**Affiliations:** 1 Addictions, Drug & Alcohol Institute, University of Washington, 1107 NE 45th. Street, Suite 120, Seattle, WA 98125 USA; 2 Department of Health Behavior and Health Education, University of Michigan, Ann Arbor, MI USA; 3 Firearm Injury & Policy Research Program, Harborview Injury Prevention & Research Center, University of Washington, Seattle, WA USA; 4 Department of Epidemiology, School of Public Health, University of Washington, Seattle, WA USA; 5 Department of Emergency Medicine, School of Medicine, University of Washington, Seattle, WA USA; 6 School of Social Work, University of Washington, Seattle, WA USA; 7 Social Development Research Group, University of Washington, Seattle, WA USA; 8 Department of Pediatrics, School of Medicine, University of Washington, Seattle, WA USA

**Keywords:** Firearm, Recruitment, Retention, System avoidance

## Abstract

**Background:**

We discuss barriers to recruitment, retention, and intervention delivery in a randomized controlled trial (RCT) of patients presenting with firearm injuries to a Level 1 trauma center. The intervention was adapted from the Critical Time Intervention and included a six-month period of support in the community after hospital discharge to address recovery goals. This study was one of the first RCTs of a hospital- and community-based intervention provided solely among patients with firearm injuries.

**Main text:**

Barriers to recruitment included limited staffing, coupled with wide variability in length of stay and admission times, which made it difficult to predict the best time to recruit. At the same time, more acutely affected patients needed more time to stabilize in order to determine whether eligibility criteria were met. Barriers to retention included insufficient patient resources for stable housing, communication and transportation, as well as limited time for patients to meet with study staff to respond to follow-up surveys. These barriers similarly affected intervention delivery as patients who were recruited, but had fewer resources to help with recovery, had lower intervention engagement. These barriers fall within the broader context of system avoidance (e.g., avoiding institutions that keep formal records). Since the patient sample was racially diverse with the majority of patients having prior criminal justice system involvement, this may have precluded active participation from some patients, especially those from communities that have been subject to long and sustained history of trauma and racism. We discuss approaches to overcoming these barriers and the importance of such efforts to further implement and evaluate hospital-based violence intervention programs in the future.

**Conclusion:**

Developing strategies to overcome barriers to data collection and ongoing participant contact are essential to gathering robust information to understand how well violence prevention programs work and providing the best care possible for people recovering from injuries.

**Trial registration:**

ClinicalTrials.govNCT02630225. Registered 12/15/2015.

## Introduction

The Helping Individuals with Firearm Injuries (HiFi) study was launched to better understand the effectiveness of hospital- and community-based programs in reducing violence and subsequent injuries during and after recovery from gunshot wounds (GSWs) (Rowhani-Rahbar et al. 2016; Lyons et al., 2021). The intervention was based on the Critical Time Intervention and included a phased intensity, with hospital and community-based interactions with the Support Specialist (Herman and Conover 2011; Tomita and Herman 2012). The study was conducted among patients presenting to Harborview Medical Center, a Level 1 trauma center in Seattle, Washington. The study is described in detail in Lyons et al. (2020), Lyons et al. (2021). The intervention goal was to support overall health and well-being, while the primary outcome was preventing arrest over two-years following hospital discharge, with a secondary outcome of preventing injury recidivism.

Intervention participants were offered six-months of support, beginning with a motivational interviewing-based goal-setting discussion, during which they met with the Support Specialist to address a broad range of recovery goals and connect to resources in the community after discharge. They were compared to people who were offered a list of referral services. Both groups had access to hospital-based social workers. The hospital did not offer a separate Hospital-Based Violence Intervention program (HVIP). Self-reported interview data were linked with Washington State administrative records documenting arrests and hospitalizations.

Several studies examined strategies for improving recruitment and retention with patients who sustain trauma. (McFarlane 2007; Clough et al. 2011; Monopoli et al. 2018). Other studies examined enrollment of people from minority racial and ethnic populations, who constituted 46% of the study sample in HiFi. Those studies point to understanding cultural context, engaging stakeholders, and being intentional in recruitment (Daunt 2003; Dancy et al. 2004; Durant et al. 2007; Heller et al. 2014). Barriers also exist more broadly in conducting RCTs, given their complexity (De Salis et al. 2008; Howard et al. 2009; Sibai et al. 2012; Thoma et al. 2010). Though considerable effort went into addressing these concerns during the study, challenges persisted.

High rates of recruitment and retention are possible in firearm injury studies, though challenges in our study were likely more systemic (Carter et al. 2015; Cunningham et al. 2015). Most participants in our sample (72.8%) had a prior arrest, pointing to a possible climate of distrust for institutions especially considering the long and sustained history of trauma and racism (Goffman 2009). At the same time, concerns regarding judgment, stigma, and loss of privacy may have dissuaded participants from help-seeking within the healthcare system (Jacoby et al. 2020). As such, participants may have engaged in “system avoidance,” avoiding institutions that keep formal records (Brayne 2014). One form of system avoidance is providing incorrect identifying and contact information to the healthcare and research staff. In our commentary we discuss the barriers we encountered during the trial and our attempts to resolve them. A summary of these barriers and our approaches to overcome them are provided in Table 1.

**Table 1:** Barriers to recruitment, retention and intervention delivery

Challenge	Description	Resolution
**Recruitment**		
Maximizing patient enrollment	• Unpredictable patient arrival times • Limited funding for full-time staff coverage	• Use of online resources for patient tracking • Flexible staff schedules • Prompt enrollment of discharged patients
Optimizing patient approach	• Working with medical care providers • Visiting friends and family • Imminent discharge	• Consultation with physicians on research staff • Consultation with medical care providers in hospital • Maintain rapport with medical care providers and visitors • Frequent check-ins • Leave brochure for patient to review
**Retention**		
Retaining unresponsive participants	• Housing instability • Frequently changing phone numbers • Limited cellular service or internet access • Poor communication affecting intervention delivery	• Frequent communication attempts • Try all available methods and contacts • Attempt to meet patients at scheduled medical visits • Support Specialist independently attempt contact • Provide incentives to help with phone service/transportation • Offer visits in the community
Retaining participants with limited time	• Good communication but low availability • Working hours conflict with research staffing times • Poor availability affecting intervention delivery	• Data collection via in-person, phone and online • Use text messages, email, and social media for contact • Contact outside of work hours (e.g., evenings and weekends) • Meet at scheduled medical appointments • Combine follow-up and intervention appointments
**Intervention Delivery**		
Connecting participants with community resources	• No limits on the areas of concern for recovery • Some needed resources unavailable (e.g., housing)	• Use well-trained staff with social work background • Community-based advisory team to help identify resources • Build rapport by focusing first on available resources • Rapport building helps with problem solving to identify other needed resources
Communication between research staff and Support Specialist	• First contact with the Support Specialist ideally in-person and in-hospital. • Options needed for patients discharging quickly • Some patients may not want face-to-face contact due to nature of injuries	• Research staff able to accurately describe intervention • Notify Support Specialist well in advance of initial approach • Flexible scheduling • Use phone-based delivery of the initial contact, if needed • Short intro video to familiarize patient with Support Specialist, if needed

## Recruitment barriers

Patients with GSWs arrived at all hours each day. Knowing when a participant was arriving did not ensure timely access to approach for enrollment. For the first 2.5 years of the study, funding only allowed for two part-time research assistants and one Support Specialist. Near the end of the study, four temporary research assistants were hired. This allowed for short-term, around the clock coverage. Initial participants may have been missed due to lack of 24/7 staff coverage. In some cases, patients were discharged while awaiting confirmation of eligibility.

Research staff made use of online records and flexible schedules to track patients prior to discharge. During enrollment, research staff consulted medical providers about possible imminent discharge in order to time recruitment. Research staff maintained good rapport with family members who may influence participation. Study brochures were provided. Patients discharged before approach were later contacted for enrollment, either using contact information from medical records, or by attempting to meet patients at scheduled outpatient appointments.

## Retention barriers

The study only required one form of direct or collateral contact information. Housing instability, including homelessness, transiency, and eviction often coincided with more challenges in communication, including unstable access to internet for emails or to cellular devices. Frequent communication attempts were time-consuming, with staff regularly maximizing the allowable number of contact attempts without successful contacts. Even participants with greater stability were challenging to retain due to work schedules. Inevitably, some participants were unreachable after their baseline visit.

Where possible, intervention visits were coupled with follow-up medical appointments to maximize subject time and help boost retention. Research staff were often able to visit participants in the field, but the tradeoff was potentially missing new admissions. For follow-up visits, data collection was done either in-person, by phone or online. We utilized text messages, email, and social media for communications, and contacted participants outside of normal work hours.

## Intervention delivery barriers

As a patient-centered intervention, there were no limits to the areas of concern that participants could identify for their recovery. Community-based professionals across different disciplines helped identify available community resources, yet some concerns were consistently hard to address (e.g., temporary housing or shelters). While many intervention participants requested help submitting claims for crime victim’s compensation, claims were sometimes delayed or denied due to perceived lack of cooperation with law enforcement, prior criminal history, or delays in police report filings. For participants who were unable to remain employed because of their injuries, these delays could be significant. The Support Specialist attempted to maintain rapport in all areas, even if an area of concern could not be fully addressed.

Research staff notified the Support Specialist in advance of initial approach. The Support Specialist made patient contact as quickly as possible, ideally in-person and during the index hospitalization to build a strong helping relationship and enhance retention. If an in-person option was not possible, phone-based introductory calls were conducted successfully. Video conferencing was also an available option. The Support Specialist focused on the issues which could be addressed in a timely manner. This would sometimes lead to participants discovering options to address longer-term problems (e.g., short-term housing).

## Conclusion

Violence is a pervasive public health problem and leading cause of morbidity and mortality in the U.S. (Sumner et al. 2015). A violent injury treated in the emergency department often represents the only access point to the healthcare system for those most at risk for violent re-injury (Rowhani-Rahbar et al. 2015; Cunningham et al. 2012; Garth et al. 2020). Up to 50% of patients injured by violence may suffer violent re-injury within 5 years following hospital discharge (Corbin et al. 2011). At the same time, the threat of violent re-injury cannot be discussed independently from factors such as systemic and institutional racism, affecting engagement and injury outcomes (Jacoby et al. 2018). By understanding the depths of the barriers that exist, we can better gauge the efforts needed to overcome them. More studies are needed to provide evidence of effectiveness for funding of HVIPs and ensure that healthcare services sufficiently meet patient needs, making trauma-informed approaches an integral part of trauma centers (Dicker 2016; Juillard et al. 2016). Follow-up retention is critical to both the intervention delivery and assessment of study outcomes, and requires constant attention to successfully implement.

## Data Availability

Data are not available for distribution.

## Abbreviations

HIFI: Helping Individuals with Firearm Injuries
GSW: Gunshot wound
